# Cycling Induced Spontaneous Coronary Artery Dissection in a Healthy Male

**DOI:** 10.1155/2018/2740513

**Published:** 2018-06-07

**Authors:** Farrukh Nadeem Jafri, David Solarz, Craig Hjemdahl-Monsen

**Affiliations:** ^1^Department of Emergency Medicine, Albert Einstein College of Medicine, White Plains Hospital, USA; ^2^Department of Cardiology, Columbia University Medical Center, White Plains Hospital, USA; ^3^Interventional Cardiology, Columbia University Medical Center, White Plains Hospital, USA

## Abstract

**Introduction:**

Spontaneous coronary artery dissection (SCAD) is a rare but important cause of acute coronary syndrome with a spectrum of disease that can include unstable angina, acute myocardial infarction, or sudden cardiac death. It has also been found in case reports to be caused by shear stress from physical exertion. We present a rare cycling induced SCAD that occurred in our institution in an otherwise healthy male with no cardiac risk factors.

**Case Presentation:**

A 36-year-old male presented to the emergency department with complaints of lightheadedness and diaphoresis after a bicycle fall. In the emergency department, he complained of feeling lightheaded and diaphoretic and having mid back pain. Patient had an ECG performed which showed lateral ST segment elevation and troponin I that was positive. A coronary angiography was subsequently performed demonstrating a spontaneous coronary artery dissection of left anterior descending coronary artery.

**Conclusion:**

SCAD is a rare cause of myocardial infarction, occurring in healthy individuals, which is rarely reported in the literature. Nearly 70% are diagnosed in postmortem studies after sudden cardiac death. Only 12 cases have been reported from activities involving physical exertion and no studies to our knowledge demonstrate this.

## 1. Introduction

Spontaneous coronary artery dissection (SCAD) is a rare but important cause of acute coronary syndrome with a spectrum of disease that can include unstable angina, acute myocardial infarction, or sudden cardiac death [1–3]. While more commonly seen in patients with atherosclerotic disease, in the postpartum period or with collagen disorders, it has also been found in rare case reports to be caused by shear stress from physical exertion [1, 2, 4]. We present a rare cycling induced SCAD that occurred in our institution in an otherwise healthy male with no cardiac risk factors.

## 2. Case

A 36-year-old male, a seasoned cyclist with no past medical history, presents to the emergency department with complaints of lightheadedness and diaphoresis after a bicycle fall. Patient was participating in a bicycle race when another rider ahead of him fell causing the patient to swerve to avoid him. Patient states that he fell on his left side and hit a tree with his right leg. Patient was wearing a helmet and did not suffer any chest or head trauma. After the fall, he felt lightheaded and diaphoretic and complained of mid back pain. Patient denied any chest pains or shortness of breath. Patient was subsequently brought to the hospital directly following the accident by ambulance.

**Figure 1:**
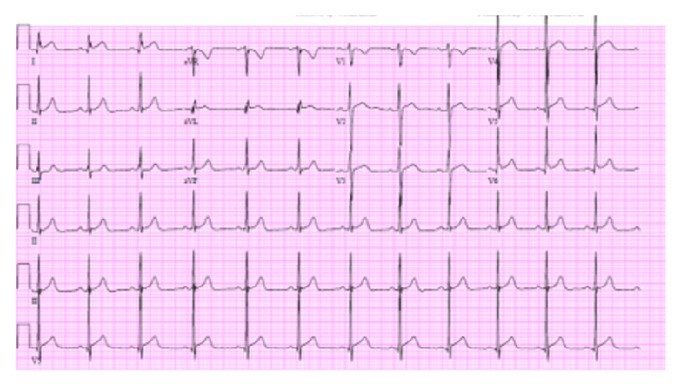
Lateral ST elevation.

In the emergency department, patient was noted to be in no acute distress; initial blood pressure was 128/69 mmHg with pulse of 65 beats per minute. He was afebrile, not tachypneic, and well appearing with marked right thigh swelling and tenderness to his medial thigh. Given the dizziness and diaphoresis initially, patient had an ECG performed which showed lateral ST segment elevation (Figure 1) and had a subsequent troponin I that was positive, 0.49ng/mL, with a Creatine Phosphokinase (CPK) of 617 U/L.

**Figure 2:**
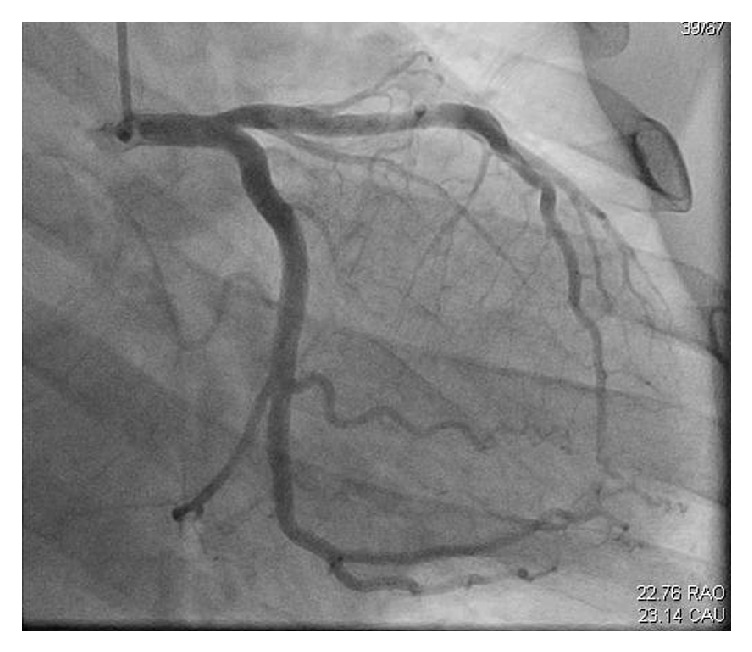
Proximal and distal lucency consistent with spontaneous LAD coronary artery dissection. The right coronary artery had a normal angiogram.

There was initial concern for a possible cardiac contusion, although the patient had no chest wall trauma and thus was admitted for further evaluation. As an inpatient, an echocardiogram was performed demonstrating normal right and left ventricular function and trace pericardial effusion while the patients troponin continued to trend upwards towards a maximum of 21ng/mL. He was loaded with Aspirin and Clopidogrel as well as initiation of a heparin infusion, Lisinopril, and a Beta Blocker. Coronary angiography was subsequently performed demonstrating a spontaneous coronary artery dissection of left anterior descending coronary artery. No further diagnostic study was performed at that time. Further history revealed that he took multiple caffeine Jello shots and drank a large cup of coffee prior to participation in the race. He denied cocaine, amphetamine, or other performance enhancing drug use (Figure 2).

The patient's CPK and troponin trended downwards on conservative medical management and his back pain resolved; therefore a stent was not placed. The patient was visiting from outside the area; discharge planning included repeat coronary angiography in 6 weeks and instructions that he will not be able to perform competitive cycling again. Should his dissection extend at that period of time or patient become symptomatic, stent placement would be considered. Patient was to continue the Aspirin and Clopidogrel until the repeat angiography was performed. Patient was discharged with plans to follow up with a cardiologist in his home state. Multiple follow-up phone calls made us unable to reach the patient and he was subsequently lost to follow-up.

## 3. Discussion

Spontaneous coronary artery dissection (SCAD) is a rare cause of myocardial infarction, commonly occurring in healthy individuals [1, 2]. Nearly 70% are diagnosed in postmortem studies after sudden cardiac death [1]. In a literature review of 440 patients found to have SCAD, 70% were female, the mean age being 42.6 [2]. A more recent single center report of 87 patients supports similar female preponderance (82%) [3].

The etiology is thought to be multifactorial, with the end result being a weakening of the arterial wall that predisposes it to dissection [4]. While many cases are found in females, particularly the postpartum period, in patients with preexisting atherosclerotic disease as well as with patients with connective tissue disorders, in men, spontaneous coronary artery dissection has been described to occur primarily after intense physical exercise or after cocaine use [1, 2, 4]. Extreme exertion at onset of SCAD was more common in males (43%) than females (2.8%) [3]. Shear stress, with or without known plaque, can be a possible explanation [1, 2, 4].

SCAD caused by physical exertion has been seen in cases of seasoned athletes training for marathons with prolonged and intense schedules [5, 6], found in a patient during weightlifting [7, 8] and playing a pick-up basketball game [9] as well as in patients who just began a new workout regimen [10]. There is only one other case study of a cyclist who developed SCAD after a marathon; however, this was a delayed presentation detected 3 months after cycling marathon [11]. Blunt chest trauma has also been associated with SCAD. Case reports demonstrate a coronary artery dissection following a sternum fracture [12] as well as a direct kick to the chest during a soccer match [13]. In both these cases, the trauma to the chest was evident in the history and exam, while the dissection was in the right coronary artery. Our patient denied blunt chest or back trauma, making this mechanism highly unlikely. His history is more consistent with SCAD as well as location of his dissection in the LAD.

SCAD may present with unstable angina, non-ST elevation myocardial infarction, ST elevation myocardial infarction, congestive heart failure, or ventricular arrhythmias. There is an increased recognition of SCAD secondary to advancements in imaging modalities such as coronary angiogram, intravascular ultrasound, and CT angiography. [14]. Of the 440 cases in the largest review of patients with SCAD, 77% of patients presented with chest pain and 64.9% of patients had ST segment elevation with the most frequent location of dissection being the LAD, 48% [2].

Currently, there are no clear guidelines on the management of treatment for SCAD. Conservative therapy may suffice when a patient is asymptomatic and coronary flow is preserved. This conservative therapy includes medical management with Aspirin, Clopidogrel, and Beta Blockers [3]. Stent placement is appropriate in the presence of ongoing refractory or recurrent ischemia [1, 3, 15]. In a literature review of 440 patients, 344 cases were diagnosed after 1990 when coronary angiography and cardiac CT were more frequently available; 21.2% of patients treated conservatively required surgical or catheter-based intervention compared to the small percentage of patients (2.5%) who were initially treated aggressively [2].

The bioresorbable vascular scaffold (BVS) was approved by the Food and Drug Administration (FDA) 2010 and has been utilized in some case studies for SCAD [16] and has been considered for young patients with SCAD [15, 17–19]. It is important to note that such use has not been validated and safety studies do demonstrate that BVS has a higher risk of thrombosis (3.5% versus 0.9%) in stent group [16]. The theoretical advantage of BVS includes the restoration of the native vessel vasomotor and adaptive shear stress allowing for late luminal enlargement and late expansive remodeling once the scaffold degrades [17].

A more recent retrospective study of 87 patients found that all patients who had an initial conservative strategy had a benign inpatient course and that a number exhibited complete resolution on repeat angiogram (mean time was 40 months). 31 (35%) patients were managed conservatively, 13 (15%) of whom had follow-up angiograms with 9 (10%) demonstrating complete or near complete resolution, 4 (4%) of whom demonstrated persistent dissection where 2 (2%) were treated with Percutaneous Coronary Intervention (PCI). Given the good outcomes detected by early conservative management, the study recommends avoidance of PCI unless patient is undergoing active ischemia. However, it is important to note that this study was a retrospective analysis with a selection of management which was not blinded and had a small sample size with less than half having repeat angiography [3]. After 1990, reports indicate that when diagnosed, SCAD has a favorable prognosis with 95% survival and a 5% recurrent rate [11].

It is unclear whether the preexercise excessive caffeine intake contributed to the patients symptoms as there is no documentation in the literature of caffeine contributing to risk for dissection.
